# Predictive model for early detection of type 2 diabetes using patients' clinical symptoms, demographic features, and knowledge of diabetes

**DOI:** 10.1002/hsr2.1834

**Published:** 2024-01-25

**Authors:** Taiwo Adetola Ojurongbe, Habeeb Abiodun Afolabi, Adesola Oyekale, Kehinde Adekunle Bashiru, Olubunmi Ayelagbe, Olusola Ojurongbe, Saddam Akber Abbasi, Nurudeen A. Adegoke

**Affiliations:** ^1^ Department of Statistics Osun State University Osogbo Nigeria; ^2^ Department of Chemical Pathology Ladoke Akintola University of Technology Ogbomoso Nigeria; ^3^ Humboldt Research Hub‐Center for Emerging and Re‐emerging Infectious Diseases Ladoke Akintola University of Technology Ogbomoso Nigeria; ^4^ Department of Medical Microbiology and Parasitology Ladoke Akintola University of Technology Ogbomoso Nigeria; ^5^ Statistics Program, Department of Mathematics, Statistics, and Physics, College of Arts and Sciences Qatar University Doha Qatar; ^6^ Statistical Consulting Unit, College of Arts and Sciences Qatar University Doha Qatar; ^7^ Melanoma Institute Australia The University of Sydney Sydney Australia

**Keywords:** demographic features and clinical symptoms, machine learning, prediction, patients' knowledge of diabetes, type 2 diabetes

## Abstract

**Background and Aims:**

With the global rise in type 2 diabetes, predictive modeling has become crucial for early detection, particularly in populations with low routine medical checkup profiles. This study aimed to develop a predictive model for type 2 diabetes using health check‐up data focusing on clinical details, demographic features, biochemical markers, and diabetes knowledge.

**Methods:**

Data from 444 Nigerian patients were collected and analysed. We used 80% of this data set for training, and the remaining 20% for testing. Multivariable penalized logistic regression was employed to predict the disease onset, incorporating waist‐hip ratio (WHR), triglycerides (TG), catalase, and atherogenic indices of plasma (AIP).

**Results:**

The predictive model demonstrated high accuracy, with an area under the curve of 99% (95% CI = 97%–100%) for the training set and 94% (95% CI = 89%–99%) for the test set. Notably, an increase in WHR (adjusted odds ratio [AOR] = 70.35; 95% CI = 10.04–493.1, *p*‐value < 0.001) and elevated AIP (AOR = 4.55; 95% CI = 1.48–13.95, *p*‐value = 0.008) levels were significantly associated with a higher risk of type 2 diabetes, while higher catalase levels (AOR = 0.33; 95% CI = 0.22–0.49, *p* < 0.001) correlated with a decreased risk. In contrast, TG levels (AOR = 1.04; 95% CI = 0.40–2.71, *p*‐value = 0.94) were not associated with the disease.

**Conclusion:**

This study emphasizes the importance of using distinct clinical and biochemical markers for early type 2 diabetes detection in Nigeria, reflecting global trends in diabetes modeling, and highlighting the need for context‐specific methods. The development of a web application based on these results aims to facilitate the early identification of individuals at risk, potentially reducing health complications, and improving diabetes management strategies in diverse settings.

## 1 INTRODUCTION

Type 2 diabetes mellitus is a metabolic condition characterized by hyperglycemia, which is caused by insufficient insulin secretion, action, or a combination of both.^1^, ^2^, ^3^ Fasting blood sugar (FBS) was first described by the American Diabetes Association as a diagnostic indicator of prediabetic conditions.^4^, ^5^, ^6^ However, this clinical stage is often overlooked because it is usually asymptomatic in the affected individuals.^6^ Several other predisposing factors have been linked to the onset of type 2 diabetes, including regular consumption of foods with a high glycaemic index,^7^ obesity, high‐density lipoprotein (HDL) and low‐density lipoprotein (LDL) levels, and hip and waist circumference measurements.^8^ Demographic features such as age and sex are also known to be associated with the development of type 2 diabetes mellitus.^8^, ^9^, ^10^

Diabetes is a chronic condition that affects millions of people globally and is projected to be one of the leading causes of noncommunicable mortality by 2030.^11^ The global burden and epidemiological trends of type 2 diabetes mellitus have been extensively documented, highlighting its growing impact on public health systems worldwide. Khan et al.^12^ provided a comprehensive overview of the global epidemiology of type 2 diabetes, emphasizing its increasing prevalence and the urgent need for effective management strategies. This perspective is particularly relevant in the context of recent health crises, such as the COVID‐19 pandemic, where Huang et al.^13^ identified a significantly increased risk of severe outcomes and mortality among hospitalized patients with diabetes in Mexico. These findings emphasize the critical need for effective diabetes management and preventive strategies. Furthermore, the complexity of type 2 diabetes as a multifactorial disease has been well articulated by Chatterjee et al.^14^ who underscored the intricate interplay of genetic, environmental, and lifestyle factors in its pathogenesis and progression.

Unnikrishnan et al.^15^ also shed light on the diabetes epidemic in India, underscoring the high prevalence of complications and pressing the need for improved healthcare responses. Type 2 diabetes not only has immediate health implications, but also exerts significant economic strain globally. A systematic review by Seuring et al.,^16^ elucidated the extensive economic costs associated with type 2 diabetes, including both direct medical costs and indirect costs such as lost productivity due to disease‐related morbidity and mortality. These studies provide a comprehensive picture of the global challenges posed by type 2 diabetes and reinforce the urgency for research efforts, such as the present study, which aims to enhance early detection and intervention, mitigate complications, and ultimately alleviate the global burden of this pervasive condition.

Nigeria is the most populous country in Africa and has a large and increasing burden of diabetes, particularly type 2 diabetes mellitus.^17^ However, since 1992, when a prevalence of 2.2% has been reported, no national health survey has been conducted to determine the prevalence and risk factors of diabetes in the country.^18^ This lack of current information on diabetes in Nigeria has hindered efforts to effectively manage the disease. Despite efforts to determine the prevalence and risk factors of diabetes in Nigeria, there are significant gaps in the country's management of the disease.^19^ The Diabetes Association of Nigeria and the Endocrine and Metabolism Society of Nigeria are responsible for developing diabetes management guidelines. However, there remain unanswered research questions and practical gaps in the country's diabetes management practices.^19^ Additionally, the sociocultural context in Nigeria influences healthcare providers' practices regarding self‐management support; however, this aspect has not been fully explored.^20^

Despite the global prevalence of type 2 diabetes, many individuals remain undiagnosed until complications arise. The prevention of acute problems and reduction in the risk of long‐term complications rely on ongoing patient awareness, early diagnosis, and self‐management. Substantial evidence supports the use of various therapies to improve outcomes.^21^ Health check‐ups are crucial for health management because of increased health awareness.^21^ These examinations provide crucial information for disease diagnosis and patient care. Regular examinations by physicians provide the opportunity for early intervention and can help detect risk factors for chronic conditions such as type 2 diabetes. However, different individuals have varying self‐care and routine medical checkup habits, and some populations have low annual health checkup compliance.^22^ This emphasizes the need for advanced predictive and cutting‐edge models to facilitate early detection and targeted intervention strategies, thereby mitigating the global impact of this chronic condition.

Recent advancements in diabetes prediction have introduced various models, each contributing uniquely to the field. Studies have employed diverse methodologies ranging from neural networks, as seen in multilayer and probabilistic models,^23^ to machine learning techniques such as the hybrid‐twin support vector machine (SVM).^24^ Other approaches include categorizing treatment plans using J48 classifiers,^25^ developing diagnostic tools that combine fuzzy logic, neural networks, and case‐based reasoning.^26^ and applying hybrid models such as kernel SVM for high‐accuracy diagnosis^27^; notably, some studies focused on lifestyle‐related risk prediction using the PIMA Indian diabetes data set,^28^ while others such as Jahani et al.^29^ and Hashi et al.^30^ emphasized neural network‐based models for disease onset and progression. Additionally, innovative techniques such as controlled binning and multiple regression.^31^ and noninvasive glucose estimation using an elastic net model.^32^ further illustrate the diverse range of predictive strategies being explored in diabetes research. Alix et al.^33^ developed a predictive model for type 2 diabetes using clinical and demographic parameters. Lai et al.^34^ developed a predictive model to identify Canadian patients at risk of diabetes using demographic data and laboratory results from medical visits. Furthermore, racial disparities were examined to assess the effectiveness of risk prediction models for incident type 2 diabetes.^35^

In line with previous diabetes research, our study adopted a comprehensive multivariate framework. This approach was designed to integrate a broader spectrum of variables, including clinical symptoms, demographic characteristics, patients' knowledge of diabetes, and biochemical data, into a predictive model. Such an integration aligns with the recent shift in diabetes research towards more sophisticated, data‐intensive models that aim to capture the multifaceted nature of the disease. The necessity of this approach has been underscored in recent literature, with studies by Collin et al.,^36^ Fregoso‐Aparisio et al.,^37^ Eyiji et al.,^38^ and Tuppad et al.^39^ highlighting the importance of incorporating multiple risk factors into predictive models for diabetes.

Furthermore, recognizing the diverse manifestations of type 2 diabetes across different populations, our study specifically focused on the Nigerian context. This study addresses a notable gap in the literature that has predominantly concentrated on Western populations. Uloko et al.,^18^ Okoro et al.,^40^ Chinenye et al.,^18^ and Fasanmade et al.^41^ highlighted the unique epidemiological and clinical characteristics of type 2 diabetes in African populations, underscoring the need for predictive models tailored to these specific demographic profiles. Our study's application of a predictive model in Nigeria not only contributes to a more global understanding of type 2 diabetes but also demonstrates the adaptability of such models to varied settings.

Hence, this study utilized health checkup data from patients at a Nigerian diabetes clinic and incorporated a range of indicators to predict the onset of diabetes. By integrating clinical symptoms, demographic features, and patients' knowledge of diabetes, we aimed to enhance the early detection and management of this condition. Our approach aligns with the increasing use of machine learning models in medical research, offering new insights into the complex interplay between the factors leading to type 2 diabetes. Such an endeavor is crucial for the early identification of high‐risk individuals. By identifying patients at high risk of developing type 2 diabetes early, healthcare professionals can provide personalized education and care to prevent complications and improve outcomes, ultimately leading to better overall health and well‐being worldwide.

## 2 METHODS

### 2.1 Study area, design, and participants

This hospital‐based case‐control study, conducted from October 2018 to March 2021, included patients diagnosed with type 2 diabetes mellitus using a convenience sampling technique.^42^ A total of 444 participants were selected based on accessibility and willingness to participate. The data included 43 characteristics, such as clinical details, demographic information, and knowledge and attitudes toward diabetes. Type 2 diabetes mellitus was diagnosed by the attending physician using the American Diabetes Association Criteria,^4^, ^5^, ^6^ with an FBS level of 126 mg/dL or higher.^21^ The control group underwent a health examination and was confirmed to be free of diabetes based on FBS levels and a well‐calibrated Accu‐Chek glucometer strip confirmatory laboratory report of fasting blood sugar and glycated hemoglobin. All the participants completed a structured questionnaire that captured their demographic information.

### 2.2 Ethical consideration

The Research Ethical Review Committee of the State Hospital, Asubiaro, situated in Osogbo, Osun State, Nigeria, granted ethical approval for this study (approval number: HREC/27/04/2015/SSO/42).

### 2.3 Inclusion and exclusion criteria

Participants aged >18 years diagnosed with type 2 diabetes were included in the study.^4^, ^5^, ^6^, ^21^ Obesity was defined as a BMI greater than 30 kg/m^2^.^43^, ^44^ Pregnant women, those with persistent alcoholism, and those with a history of hepatitis were excluded.^43^, ^44^

### 2.4 Outcomes

The endpoint of this study was to identify patients with type 2 diabetes mellitus. Patients with FBS levels ≥126 mg/dL were grouped into those with confirmed diabetes, and those below 126 mg/dL were labeled as nondiabetic.

### 2.5 Features

Several potential biomarkers of type II diabetes have been identified, including biochemical and clinical parameters,^45^ demographic characteristics, and patients' knowledge and attitudes towards the disease.^46^ The biochemical and clinical parameters used in the study include apolipoprotein C‐III (APO‐CIII), systolic blood pressure (SysBp), diastolic blood pressure (DiaBp), hypertensive status, hypertensive group, waist circumference (WC), Hip circumference (HC), waist‐hip ratio (WHR), total cholesterol (TC), triglyceride (TG), high‐density lipoprotein cholesterol (HDL‐c), low‐density lipoprotein cholesterol (LDL‐c), atherogenic indices of plasma (AIP), cardiac risk ratio (CRR), non‐high‐density lipoprotein cholesterol (Non‐HDL‐c), atherogenic coefficient, malondialdehyde (MDA), superoxide dismutase (SOD), catalase, body mass index (BMI), carbohydrate counting (CHO), hemoglobin A1C (HbA1C), retinopathy, nephropathy, feet neuropathy, heart attack, slowed digestion, gastroparesis and hypertensive status. Demographic features included the sex and age of the patients, and the study also considered questions about the knowledge and attitudes of patients towards diabetes, as well as other factors that have been reported in the literature.^46^, ^47^ Details regarding the measurements of the biochemical and clinical parameters used in the study and their units are provided in Supplementary Table 1.

## 3 STATISTICAL ANALYSIS

### 3.1 Baseline patient characteristics

This study included 444 patients, with 312 and 132 in the training and test sets, respectively. Patients' baseline characteristics were summarized using frequencies and proportions for categorical variables and medians and ranges for continuous variables. The study compared baseline characteristics between patients with and without type 2 diabetes using the Wilcoxon rank‐sum test for continuous variables and Pearson's chi‐square test for categorical variables, with Yates' continuity correction when appropriate. Continuous variables in the training set were scaled to have a mean of zero and standard deviation of one. The variables of the test set were mapped to the relevant variables in the training cohort. Univariate logistic regression models were used to assess the relationship between each component and type of diabetes as well as the association between the risk of type 2 diabetes and the patients' clinical, demographic, and knowledge of diabetes. A multivariate penalized logistic model implemented under stratified nested cross‐validation for parameter optimization and sequential backward feature selection was used to predict the risk of type 2 diabetes.

### 3.2 Model cross‐validation

Nested cross‐validation was performed on the training data set using two levels of stratified cross‐validation involving inner and outer folds to obtain good classification accuracy and prevent overfitting. The model parameters were optimized, and informative feature subsets were determined in the inner folds, while the best (inner) model performance was assessed in the outer fold. For the outer fold, the training data set was split into 10‐fold cross‐validation (CV), with one‐fold kept as a test set, and the remaining nine folds were split into ten stratified folds, nine folds for model training, and the remaining fold for the test set, to provide an unbiased evaluation of the model fit on the inner training set while tuning the model's hyperparameters and selecting optimal features. Twenty repetitions of the outer and inner folds were performed to obtain a robust model; the outer and inner folds were also stratified to correct the imbalance in the data set.

### 3.3 Optimal feature selection and hyperparameters

Sequential backward selection was employed for feature selection, starting with the utilization of all features and eliminating non‐informative features in each iteration to enhance the performance of the model. This process was continued until no further improvements were observed. Once the optimal combination of hyperparameter and feature subsets was identified to maximize the performance metrics in the test set, the model was retrained on the outer training set and tested on the test set from the outer CV. Subsequently, the feature subsets from all outer folds were combined using a voting strategy that retained features with a frequency of more than 50% in all outer folds as informative; these features were chosen as the final feature subset. The median of the best hyperparameters from the outer CV folds was used to fit the final model. Finally, summary performance estimates were generated by averaging the area under the curve (AUC) of the receiver operating characteristic (ROC) curve.

### 3.4 Performance evaluation

To generate summary performance estimates, we averaged the AUC of the ROC curve and other performance evaluations such as sensitivity, specificity, positive predictive value (PPV), and negative predictive value (NPV) of the CV. The sensitivity $\left(\frac{\mathrm{TP}}{\mathrm{TP}+\mathrm{FP}}\right)$, specificity $\left(\frac{\mathrm{TN}}{\mathrm{TN}+\mathrm{FN}}\right)$, PPV $\left(\frac{\mathrm{TP}}{\mathrm{TP}+\mathrm{FN}}\right)$ and NPV $\left(\frac{\mathrm{TN}}{\mathrm{TN}+\mathrm{FP}}\right)$, where TP, FP, TN, and FN are the numbers of true positives, false positives, true negatives, and false negatives, respectively, were calculated using the default cutoff value (0.5) for the positive or negative diabetic class. Model parameter values were chosen to maximize the predicted positive class. All statistical analyses were performed in R, and the final model was developed using the *caret* library (version 6.0.93). The final model's receiver operating characteristic (ROC) curve was drawn using the *pROC* library (version 1.18.0). Statistical significance was set at *p*‐value < 0.05.

## 4 RESULTS

### 4.1 Patient characteristics

The training set included 312 individuals, including 172 and 140 with and without type 2 diabetes, respectively. There was no significant difference in the prevalence of obesity between the participants with and without diabetes (48.5% vs. 54.4%, respectively; *p*‐value = 0.36). However, a marked difference was observed in the use of diabetes medication, with 100% of participants without diabetes not on medication compared with only 6% of participants with diabetes not on medication (*p*‐value < 0.001). The median age of the patients was 40 years (Table 1). There was no significant difference in the age of the participants with and without diabetes (median (range): 40.0 (21.0–68.0) versus 41.0 (21.0–65.0); *p*‐value = 0.86). Female patients with diabetes had a significantly higher incidence of diabetes than male patients (84.6% vs. 15.4%, *p*‐value = 0.008).

**Table 1:** Patients clinical, demographical and knowledge of diabetes characteristics in the training set.

Predictor	No (*N* = 130)	Yes (*N* = 182)	Total (*N* = 312)	*p* value
**Obese**				0.36
No	63 (48.5%)	99 (54.4%)	162 (51.9%)	
Yes	67 (51.5%)	83 (45.6%)	150 (48.1%)	
**Are you on any medication for diabetes**				**<0.001**
No	130 (100.0%)	11 (6.0%)	141 (45.2%)	
Yes	0 (0.0%)	171 (94.0%)	171 (54.8%)	
**Age**				0.86
Median (Range)	41.0 (21.0, 65.0)	40.0 (21.0, 68.0)	40.0 (21.0, 68.0)	
**Sex**				**0.008**
Female	93 (71.5%)	154 (84.6%)	247 (79.2%)	
Male	37 (28.5%)	28 (15.4%)	65 (20.8%)	
**BW**				0.62
Median (Range)	74.0 (48.0, 95.0)	70.0 (48.0, 95.0)	71.0 (48.0, 95.0)	
**Height**				0.30
Median (Range)	1.60 (1.40, 1.79)	1.590 (1.30, 1.86)	1.590 (1.30, 1.86)	
**BMI**				0.76
Median (Range)	31.2 (17.8, 40.16)	26.74 (18.370, 43.79)	27.85 (17.76, 43.79)	
**APO‐CIII**				**0.02**
Median (Range)	8.88 (4.080, 80.48)	9.800 (1.40, 18.0)	9.43 (1.40, 80.48)	
**SysBp**				0.88
Median (Range)	127.0 (93.0, 240.0)	129.5 (90.0, 240.0)	128.0 (90.0, 240.0)	
**DiaBp**				0.38
Median (Range)	80.0 (56.0, 104.0)	84.0 (56.0, 127.0)	82.0 (56.0, 127.0)	
**Hypertensive status**				0.85
Hypertensive	44 (33.8%)	65 (35.7%)	109 (34.9%)	
Normal	51 (39.2%)	73 (40.1%)	124 (39.7%)	
Pre hypertensive	35 (26.9%)	44 (24.2%)	79 (25.3%)	
**Hypertensive Group**				0.83
Hypertensive	44 (33.8%)	65 (35.7%)	109 (34.9%)	
Non hypertensive	86 (66.2%)	117 (64.3%)	203 (65.1%)	
**WC**				0.09
Median (Range)	85.95 (62.66, 107.23)	86.71 (63.10, 126.71)	86.71 (62.66, 126.71)	
**HC**				0.42
Median (Range)	98.22 (75.220, 118.79)	96.46 (70.65, 136.27)	97.65 (70.65, 136.27)	
**WHR**				**<0.001**
Median (Range)	0.88 (0.83, 0.90)	0.91 (0.88, 0.93)	0.90 (0.83, 0.93)	
**CHO**				**<0.001**
Median (Range)	4.72 (2.63, 7.29)	5.01 (3.35, 7.13)	4.790 (2.630, 7.290)	
**TG**				**<0.001**
Median (Range)	0.84 (0.17, 3.03)	1.51 (0.61, 3.58)	1.37 (0.17, 3.58)	
**HDL‐C**				0.27
Median (Range)	1.11 (0.51, 2.28)	1.15 (0.41, 1.64)	1.13 (0.41, 2.28)	
**LDL‐C**				0.23
Median (Range)	3.11 (1.12, 5.29)	3.13 (1.72, 5.28)	3.11 (1.12, 5.29)	
**LDL‐HDL**				0.14
Median (Range)	2.89 (0.58, 7.45)	3.02 (1.10, 11.12)	2.97 (0.58, 11.12)	
**AIP**				**<0.001**
Median (Range)	−0.11 (−0.86, 0.52)	0.15 (−0.29, 0.67)	0.08 (−0.86, 0.67)	
**CRR**				**0.002**
Median (Range)	4.37 (1.76, 9.49)	4.85 (2.52, 13.80)	4.58 (1.76, 13.80)	
**NonHDL**				**<0.001**
Median (Range)	3.62 (1.24, 5.90)	3.94 (2.50, 6.13)	3.76 (1.24, 6.13)	
**Atherogenic coefficient**				**0.002**
Median (Range)	3.37 (0.76, 8.49)	3.85 (1.52, 12.80)	3.58 (0.76, 12.80)	
**MDA**				**<0.001**
Median (Range)	0.60 (0.33, 1.15)	0.830 (0.10, 1.68)	0.730 (0.10, 1.68)	
**SOD**				**<0.001**
Median (Range)	166.03 (23.08, 310.15)	112.8 (77.86, 274.82)	132.72 (23.08, 310.15)	
**Catalase**				**<0.001**
Median (Range)	37.74 (23.50, 46.75)	23.98 (17.40, 54.43)	33.46 (17.40, 54.43)	
**HbA1c**				0.75
Median (Range)	8.51 (3.87, 14.05)	8.300 (3.90, 12.34)	8.330 (3.87, 14.05)	
**Eyes retinopathy**				0.14
No	128 (98.5%)	172 (94.5%)	300 (96.2%)	
Yes	2 (1.5%)	10 (5.5%)	12 (3.8%)	
**Kidney's proteinuria or nephropathy**				0.85
No	125 (96.2%)	173 (95.1%)	298 (95.5%)	
Yes	5 (3.8%)	9 (4.9%)	14 (4.5%)	
**Nerves or feet neuropathy**				0.89
No	120 (92.3%)	166 (91.2%)	286 (91.7%)	
Yes	10 (7.7%)	16 (8.8%)	26 (8.3%)	
**Heart attack or blocked heart arteries**				0.83
No	121 (93.1%)	167 (91.8%)	288 (92.3%)	
Yes	9 (6.9%)	15 (8.2%)	24 (7.7%)	
**Slowed digestion (gastroparesis)**				>0.99
No	117 (90.0%)	164 (90.1%)	281 (90.1%)	
Yes	13 (10.0%)	18 (9.9%)	31 (9.9%)	
**Eating too much sugar and sweet foods is a cause of Diabetes**				0.30
No	6 (4.6%)	15 (8.2%)	21 (6.7%)	
Yes	124 (95.4%)	167 (91.8%)	291 (93.3%)	
**A common cause of diabetes is insulin resistance in the body**				0.50
No	6 (4.6%)	13 (7.1%)	19 (6.1%)	
Yes	124 (95.4%)	169 (92.9%)	293 (93.9%)	
**Diabetes is hereditary**				0.47
No	18 (13.8%)	32 (17.6%)	50 (16.0%)	
Yes	112 (86.2%)	150 (82.4%)	262 (84.0%)	
**Medication is more important than diet and exercise to control diabetes**				0.24
No	32 (24.6%)	57 (31.3%)	89 (28.5%)	
Yes	98 (75.4%)	125 (68.7%)	223 (71.5%)	
**Diabetes often causes poor circulation**				0.18
No	103 (79.2%)	156 (85.7%)	259 (83.0%)	
Yes	27 (20.8%)	26 (14.3%)	53 (17.0%)	
**Cuts and wounds heal more slowly in diabetics**				0.98
No	34 (26.2%)	49 (26.9%)	83 (26.6%)	
Yes	96 (73.8%)	133 (73.1%)	229 (73.4%)	
**Diabetes can lead to decreased sensitivity of the hands fingers and feet**				0.80
No	36 (27.7%)	54 (29.7%)	90 (28.8%)	
Yes	94 (72.3%)	128 (70.3%)	222 (71.2%)	
**Tremors and sweating are signs of high sugar in the blood**				**0.01**
No	33 (25.4%)	72 (39.6%)	105 (33.7%)	
Yes	97 (74.6%)	110 (60.4%)	207 (66.3%)	
**Smoking and consumption of alcohol contributes to the complication of diabetes**				0.07
No	16 (12.3%)	38 (20.9%)	54 (17.3%)	
Yes	114 (87.7%)	144 (79.1%)	258 (82.7%)	

Further analysis revealed that patients with diabetes had significantly higher levels of various biomarkers. For instance, the levels of AIP, CRR, non‐HDL‐c, atherogenic coefficient, MDA, WHR, CHO, TG, and APO‐CIII were all significantly elevated in patients with diabetes (*p*‐value < 0.001 for AIP, non‐HDL, MDA, WHR, CHO, and TG; *p*‐value = 0.002 for CRR and atherogenic coefficient; *p*‐value = 0.02 for APO‐CIII). Conversely, the antioxidant enzymes SOD and catalase were found to be significantly lower in these patients (*p*‐value < 0.001 for both). Additionally, a higher proportion of individuals with diabetes were found to be taking glucose‐lowering medications (100% of diabetic participants vs. 6% of nondiabetic participants; *p*‐value < 0.001). In contrast, our analysis did not reveal significant associations between diabetes status and a range of clinical features such as body weight, height, BMI, SysBP, DiaBP, hypertension status, WC, HC, HDL‐c, LDL‐c, HbAlC, retinopathy, nerve or foot neuropathy, heart attack, or slow digestion.

Regarding beliefs about diabetes risk factors, individuals who did not acknowledge the roles of obesity, overeating, sugar‐ and fat‐rich diets, insulin resistance, and heredity in the development of diabetes exhibited a higher incidence of diabetes. However, these differences were not statistically significant (*p*‐value = 0.30 for obesity, overeating, sugar‐ and fat‐rich diets; *p*‐value = 0.50 for insulin resistance; *p*‐value = 0.47 for heredity). Similarly, the belief that medication is more important than diet and exercise for diabetes control was not significantly associated with the incidence of diabetes (*p*‐value = 0.24). Additionally, patients with diabetes were slightly more inclined to disbelieve that smoking and alcohol consumption contribute to disease complications, although this association approached significance (*p*‐value = 0.07). Finally, the baseline characteristics of the patients in the training and test sets were similar (Table 2).

## 5 MODELS

### 5.1 Univariate analysis

We first conducted a univariable analysis on the training set to investigate the association between each factor and diabetic status (Table 2). Increased odds ratios (OR) for patients with diabetes were associated with WC (OR = 1.26, 95% confidence interval (CI) = 1.00–1.59, *p*‐value = 0.046), WHR (OR = 205.79, 95% CI = 53.02–798.76, *p*‐value < 0.001), CHO (OR = 1.59, 95% CI = 1.25–2.03, *p*‐value = 0.002), TG (OR = 7.02, 95% CI = 4.48–11.00, *p*‐value < 0.001), AIP (OR = 6.36, 95% CI = 4.05–10.00, *p*‐value < 0.001), CRR (OR = 1.60, 95% CI = 1.21–2.12, *p*‐value = 0.001), non‐HDL (OR = 1.67, 95% CI = 1.31–2.14, *p*‐value < 0.001), atherogenic coefficient (OR = 1.60, 95% CI = 1.21–2.12, *p*‐value = 0.001), and MDA (OR = 2.42, 95% CI = 1.78–3.30, *p*‐value < 0.001). In contrast, a decreased OR for diabetes was associated with SOD (OR = 0.46, 95% CI = 0.36–0.6, *p*‐value < 0.001) or catalase (OR = 0.13, 95% CI = 0.09–0.02, *p*‐value < 0.001). The odds of diabetes were not associated with age, BMI, APO‐CIII, SysBP, DiaBP, hypertension status, WC, HC, HDL‐c, LDL‐c, HbA1C, retinopathy, nephropathy, foot neuropathy, blocked arteries, and gastroparesis, believing that eating too much sugar is a cause of diabetes, believing that diabetes is hereditary, believing that diabetes causes poor circulation, believing that cuts and wounds heal more slowly in diabetic patients, and believing that diabetes can lead to decreased sensitivity of the hands, fingers, and feet.

**Table 2:** Odd ratios (AOR) from the univariable penalized logistic regression model.

Predictor	OR	95% CI	*p* values
**Obese**			
No	1		
Yes	0.79	0.502–1.238	0.30
**Are you on any medication for diabetes**			
No	1		
Yes	10099055218	0 – Inf	0.99
**Age**	1.08	0.862–1.356	0.50
**Sex**			
Female	1.00		
Male	0.46	0.26–0.8	**0.006**
**BW**	0.97	0.78–1.22	0.82
**Height**	0.88	0.7–1.1	0.27
**BMI**	1.05	0.84–1.31	0.68
**APO–CIII**	0.95	0.76–1.19	0.64
**SysBp**	1.04	0.83–1.3	0.74
**DiaBp**	1.10	0.88–1.38	0.40
**Hypertensive status**			
Hypertensive	1.00		
Normal	0.97	0.57–1.64	0.91
Pre hypertensive	0.85	0.47–1.53	0.60
**Hypertensive group**			
Hypertensive	1.00		
Non hypertensive	0.92	0.57–1.48	0.73
**WC**	1.26	1–1.59	**0.046**
**HC**	0.94	0.75–1.18	0.61
**WHR**	205.79	53.02–798.76	**<0.001**
**CHO**	1.59	1.25–2.03	**0.002**
**TG**	7.02	4.48–11	**<0.001**
**HDL–C**	0.91	0.73–1.15	0.43
**LDL–C**	1.19	0.95–1.49	0.14
**LDL–HDL**	1.28	1–1.63	0.05
**AIP**	6.36	4.05–10	**<0.001**
**CRR**	1.60	1.21–2.12	**0.001**
**Non–HDL**	1.67	1.31–2.14	**<0.001**
**Atherogenic coeffient**	1.60	1.21–2.12	**0.001**
**MDA**	2.42	1.78–3.3	**<0.001**
**SOD**	0.46	0.36–0.6	**<0.001**
**Catalase**	0.13	0.09–0.2	**<0.001**
**HbA1c**	0.93	0.74–1.16	0.52
**Eyes mretinopathy**			
No	1.00		
Yes	3.72	0.8–17.28	0.09
**Kidney proteinuria or nephropathy**			
No	1.00		
Yes	1.30	0.43–3.97	0.65
**Nerves or feet neuropathy**			
No	1.00		
Yes	1.16	0.51–2.64	0.73
**Heart attack or blocked heart arteries**			
No	1.00		
Yes	1.21	0.51–2.85	0.67
**Slowed digestion (Gastroparesis)**			
No	1.00		
Yes	0.99	0.47–2.09	0.97
**Eating too much sugar and sweet foods is a cause of Diabetes**			
No	1.00		
Yes	0.54	0.2–1.43	0.21
**A common cause of diabetes is insulin resistance in the body**			
No	1.00		
Yes	0.63	0.23–1.7	0.36
**Diabetes is hereditary**			
No	1.00		
Yes	0.75	0.4–1.41	0.38
**medication is more important than diet and exercise to control diabetes**			
No	1.00		
Yes	0.72	0.43–1.19	0.20
**Diabetes often causes poor circulation**			
No	1.00		
Yes	0.64	0.35–1.15	0.16
**Cuts and wounds heal more slowly in diabetics**			
No	1.00		
Yes	0.96	0.58–1.6	0.88
**Diabetes can lead to decreased sensitivity of the hands fingers and feet**			
No	1.00		
Yes	0.91	0.55–1.49	0.70
**Tremors and sweating are signs of high sugar in the blood**			
No	1.00		
Yes	0.52	0.32–0.85	**0.009**
**S=Smoking and consumption of alcohol contributes to the complicationofdiabetes**			
No	1.00		
Yes	0.53	0.28–1	0.05

### 5.2 Multivariable analysis

**Figure 1:**
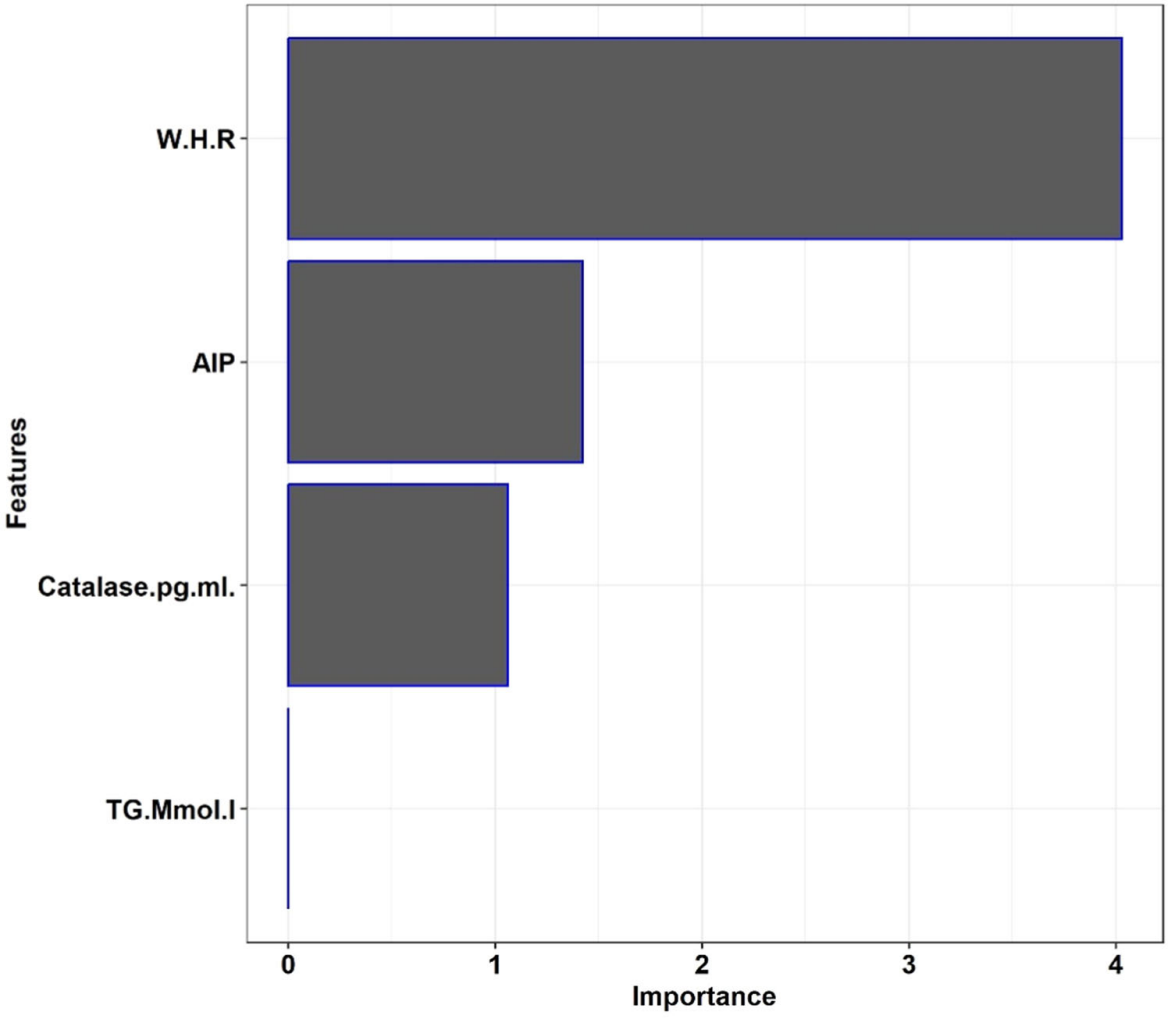
Important features of the penalized logistic regression model.

**Figure 2:**
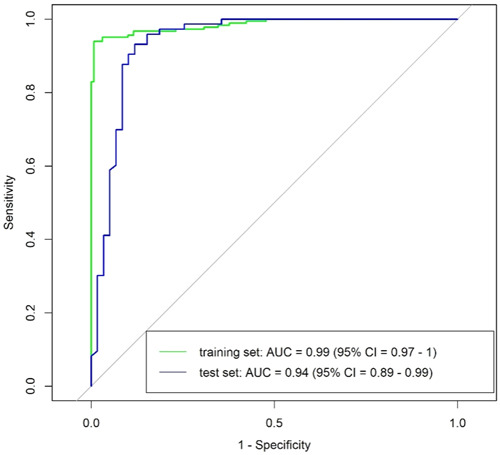
ROC curves from the penalized logistic regression model for the training (*green*) and test (blue) sets.

Factors for which the univariate odds ratio was statistically significant were included as inputs in the multivariable logistic regression model. From these variables, the final predictive model was built using nested cross‐validation, and included WHR, catalase, TG, AIP, and SOD as informative features (Figure 1), with training and test AUC values of 99% (95% CI = 97%–100%) and 94% (95% CI = 89%–99%), respectively (Figure 2). The model training and test set accuracies were 95% and 91%, respectively. The sensitivity, specificity, PPV, and NPV of the model for the training and test datasets are presented in Table 3.

**Table 3:** Performance of the penalized logistic regression model for training and test sets.

Cohort	Sensitivity	Specificity	PPV	NPV
Training set	0.95	0.95	0.93	0.97
Test set	0.85	0.96	0.94	0.89

### 5.3 Association between positive diabetes and model‐selected predictors

Table 4 presents the adjusted odds ratios (AOR), 95% CI, and p‐values of the predictors from the final multivariable model. As shown in the table, a significant increase in the AOR of type 2 diabetes was observed with WHR (AOR = 70.35; 95% CI = 10.04–493.1, *p*‐value < 0.001) and AIP (AOR = 4.55; 95% CI = 1.48–13.95, *p*‐value = 0.008). Meanwhile, TG had an increased AOR of 1.04 for type 2 diabetes (95% CI = 0.4–2.71), although this association was not statistically significant (*p*‐value = 0.94). Conversely, catalase exhibited a decreased AOR for type 2 diabetes (AOR = 0.33; 95% CI = 0.22–0.49, *p*‐value < 0.001).

**Table 4:** AOR from the multivariate penalized logistic regression model.

Predictors	AOR	95% CI	p‐values
**WHR**	70.35	10.04–493.1	<**0.001**
**TG**	1.04	0.40–2.71	0.94
**Catalase**	0.33	0.22–0.49	<**0.001**
**AIP**	4.55	1.48–13.95	**0.008**

### 5.4 Web‐based application for the prediction of type II diabetes

Our study presents a web‐based application that uses the final multivariable model to enable the early prediction of type 2 diabetes. Available at https://iv3p9h-nurudeen-adegoke.shinyapps.io/Diabetic/, the application provides personalized risk scores for developing diabetes. By identifying individuals at risk early, the tool can facilitate targeted interventions and lifestyle modifications to prevent or delay the onset of diabetes. Our app was validated and achieved high accuracy in predicting diabetes risk, making it a valuable tool for managing diabetes risk for both individuals and healthcare professionals.

## 6 DISCUSSION

We used routinely collected sociodemographic data, clinical symptoms, and patients' knowledge of diabetes to estimate the risk of developing diabetes. Our predictive model, based on multivariable penalized logistic regression, achieved an AUC of 99% (95% CI = 97%–100%) for the training set and 94% (95% CI = 89%–99%) for the test set. The model included key features, such as waist‐hip ratio (WHR), triglycerides (TG), catalase, and atherogenic indices of plasma (AIP).

Our univariate analysis revealed an association between the incidence of diabetes and certain clinical symptoms, such as WC (OR = 1.26, 95% CI = 1.00–1.59, *p* = 0.046), WHR (OR = 205.79, 95% CI = 53.02–798.76, *p* < 0.001), CHO (OR = 1.59, 95% CI = 1.25–2.03, *p* < 0.001), TG (OR = 7.02, 95% CI = 4.48–11.00, *p* < 0.001), AIP (OR = 6.36, 95% CI = 4.05–10.00, *p* < 0.001), CRR (OR = 1.60, 95% CI = 1.21–2.12, *p* = 0.001), non‐HDL‐c (OR = 1.67, 95% CI = 1.31–2.14, *p* < 0.001), atherogenic coefficient (OR = 1.60, 95% CI = 1.21–2.12, *p* = 0.001), MDA (OR = 2.42, 95% CI = 1.78–3.30, *p* < 0.001) and catalase (OR = 0.13, 95% CI = 0.09–0.02, *p* < 0.001). These associations are consistent with previous findings,^48^, ^49^ confirming an association between type 2 diabetes and these clinical symptoms.

The association between type 2 diabetes mellitus and WC or WHR has been established in previous studies.^50^, ^51^, ^52^, ^53^ This relationship is supported by the fact that central obesity, as measured by WC or WHR, produces diabetogenic substances that contribute to progression of diabetes.^52^ Seidell et al.^50^ posited that the ratio of waist circumference to hip circumference is a significant predictor of the prevalence of diabetes mellitus in adult men and women, and abdominal computed tomography (CT) scan measurements of subcutaneous fat were less significantly associated with the accumulation of intra‐abdominal (visceral) fat than the waist‐to‐hip circumference ratio. Therefore, it is important for patients to maintain a normal waist circumference and balanced waist‐to‐hip ratio to reduce their risk of developing type 2 diabetes mellitus.

Our findings are in line with those of several studies that have established associations between type 2 diabetes and CHO, AIP, non‐HDL cholesterol, AC, or TG.^54^, ^55^, ^56^ Type 2 diabetes is often accompanied by dyslipidaemia and abnormal accumulation of lipids in the bloodstream.^57^ Elevated levels of triglycerides, APO‐CIII, non‐HDL cholesterol, and atherogenic coefficient are indicative of dyslipidaemia. While a genetic predisposition has been linked to the risk of type 2 diabetes,^57^, ^58^ previous studies have also associated this condition with metabolic indicators, such as CHO, AIP, and CRR.^59^ Additionally, FBS, blood pressure, and lipid profiles, including CHO, AIP, and CRR, have been correlated with the Indian Diabetes Risk Score (IDRS).^60^ Type 2 diabetes is frequently characterized by high CHO and AIP levels, which increases the risk of cardiovascular disease.^61^

Additionally, the associations between the risk of type 2 diabetes, MDA, SOD, and catalase levels were consistent with those reported in a previous study.^62^ Hyperglycemia, a defining feature of oxidative stress and MDA diabetes, is a leading cause of type II diabetes.^63^ The development of diabetic complications such as cardiovascular disease (CVD) is heavily influenced by oxidative stress.^64^ In addition, the relationship between type 2 diabetes and SOD activity has been examined in several studies. SOD is a vital antioxidant enzyme that protects cells against oxidative stress.^65^ Patients with type 2 diabetes have been shown to have lower SOD activity,^65^ a sign of higher oxidative stress, and catalase overexpression lowers the expression of angiotensinogen and apoptosis in diabetic mice.^66^ Oxidative stress is a major contributor to diabetes complications such as CVD. Reducing oxidative stress and maintaining SOD, catalase, and MDA activities may be crucial components of type 2 diabetes care to reduce the risk of diabetic complications.

Numerous studies have demonstrated that high FBS levels are necessary for transition from a healthy state to diabetes mellitus.^67^, ^68^ Yeboah et al.^69^ claimed that FBS is a distinct risk factor for the emergence of type 2 diabetes, implying that efforts to lower the FBS incidence will also reduce the incidence of type 2 diabetes mellitus in the long run. Therefore, the incidence of diabetes mellitus can be effectively controlled through early diagnosis and FBS treatment. Our study did not find significant associations between diabetes status and clinical features such as body weight, height, BMI, SysBP, DiaBP, hypertension status, WC, HC, HDLC, LDLC, HbAlC, retinopathy, nerve or foot neuropathy, heart attack, or slow digestion. This indicates that although these clinical features are commonly associated with diabetes, they may not be definitive indicators of the disease in all cases.

In addition to the established factors for diabetes prediction, our study incorporated an assessment of patients' knowledge and attitudes towards the disease. Our findings revealed a general deficit in the understanding of the risk factors associated with type 2 diabetes among the study participants. This lack of awareness may contribute to the prevalence of the disease, particularly in developing nations, where attitudes towards diabetes and its symptoms tend to be more dismissive. Interestingly, our analysis revealed that individuals who did not recognize the contribution of factors such as obesity, overeating, sugar‐ and fat‐rich diets, insulin resistance, and heredity to the development of diabetes exhibited a higher incidence of the disease. However, these associations were not statistically significant, suggesting a potential gap in the awareness or even denial of these risk factors among individuals with diabetes. In light of these findings, we recommend re‐evaluation of strategies to combat diabetes, with a primary focus on promoting awareness of its risk factors. Educating the public and fostering appropriate attitudes towards diabetes prevention are essential steps towards maintaining a diabetes‐free society.

Additionally, the belief that medication is more important than diet for controlling diabetes did not show a significant association with the incidence of diabetes. This finding underscores the complexity of diabetes management and the need for comprehensive patient education. Unexpectedly, our analysis indicated that individuals taking glucose‐lowering medications had a higher risk of developing type 2 diabetes. This finding may initially seem counterintuitive as these medications are typically prescribed for diabetes management. However, it is important to consider that many patients may not commence medication until disease progression, which could reflect a more advanced stage of diabetes at the time of diagnosis. This underscores the crucial role of early detection and intervention in the management of type 2 diabetes. Further research is needed to understand the causal relationships and the underlying mechanisms of this association. Our data also suggest a slightly higher tendency among patients with diabetes to not believe that smoking and alcohol consumption contribute to diabetic complications. This further highlights the importance of patient education on lifestyle factors in diabetes management.

Another important aspect of our study was the use of machine learning techniques to predict the occurrence of diabetes using routinely collected data. By utilizing multivariate penalized logistic regression implemented under nested cross‐validation with sequential backward feature selection, our model maximized its predictive power while minimizing the risk of overfitting. This data‐driven approach facilitates the identification of key predictors of type 2 diabetes and provides a more precise prediction of diabetes risk at the individual level. Although the application of machine learning techniques in this context is not novel, it demonstrates the potential of such models to enhance clinical decision‐making and resource allocation, particularly in resource‐limited settings. This careful application of machine learning has the potential to transform the way healthcare professionals approach diabetes control and treatment, ultimately improving patient outcomes and efficiency of the healthcare system.

Additionally, the web‐based application presented in this study harnesses power predictive analytics to provide a practical approach for early detection and management of type 2 diabetes. The application was developed using a well‐validated model with the potential to revolutionize preventative healthcare strategies. The application's ability to deliver personalized risk scores not only empowers individuals with crucial information about their health, but also assists healthcare professionals in providing timely and targeted interventions. This optimization of healthcare resources has the potential to mitigate diabetes epidemics. In Nigerian healthcare settings, the deployment of the proposed application has the potential to be transformative. Given the country's diverse and widespread population, healthcare access remains uneven. This application can help bridge the disparity between urban and rural healthcare delivery by facilitating remote screening, enabling individuals in less accessible areas to assess their health risks without immediate clinic visits.

Furthermore, the app can be integrated into Nigeria's primary health care system as a preliminary screening tool for general practitioners. By promptly identifying high‐risk patients, clinicians can recommend specialized care or more frequent monitoring, directing resources to those who need them the most. With the increasing mobile phone penetration in Nigeria, optimizing the application for mobile use can further extend its reach. However, challenges, such as enhancing user digital literacy and ensuring consistent Internet connectivity, remain. Initiatives for user education and collaboration with telecommunication companies to explore potential incentives or data bundles should be pursued to encourage app usage. A significant advantage of the proposed application is its private‐first approach. The app provides essential insights without storing any personal patient data, ensuring user privacy and reducing concerns regarding data misuse. This design choice reflects our commitment to user trust and ethical deployment of digital health tools.

Although our study offers valuable insights, it has certain limitations that must be acknowledged. The convenience sampling utilized in our research may have resulted in selection bias, thereby restricting the generalizability of our findings beyond the study population. The study was conducted at a single hospital in Osogbo, Osun State, Nigeria and may not be representative of other geographical or sociocultural contexts. Although effective in elucidating associations, a case‐control design is inadequate for establishing causality. Additionally, reliance on structured questionnaires may have introduced recall bias due to potential misreporting by the participants. Exclusion of specific groups, such as pregnant women and those with persistent alcoholism or a history of hepatitis, could limit the applicability of our findings to these segments. Relying solely on FBS levels for diabetes diagnosis, despite its reliability, may overlook certain borderline or alternative diagnostic cases. Finally, potential unmeasured confounders and variability in biochemical and clinical measurements may have influenced the study outcomes. Notwithstanding these limitations, our findings provide a basis for further comprehensive investigation on this topic.

In conclusion, we have successfully developed a highly accurate predictive model that can aid in the early identification and management of diabetes. The AUC obtained in our study was better than 88% reported by Walford et al.^70^ In addition, our predictive model demonstrated robust performance with training and test set accuracies of 95% and 91%, respectively, showing a notable advantage in terms of accuracy and reliability compared to similar studies in the field. For instance, while the model in Anand et al.^28^ achieved a commendable 75% accuracy using the PIMA data set and CART classifier, our model surpassed this, indicating a higher predictive precision. Similarly, Jakhmola et al.^31^ and Anand et al.^28^ reported accuracies of 77.85% and 75%, respectively, which were significantly lower than the performance of our model. Notably, our model even outperformed the 78.17% accuracy achieved by the decision tree model ^71^ and the 70.8% accuracy of the treatment classification model.^25^ Moreover, while the expert healthcare system^30^ achieved a high accuracy of 90.43%, our model slightly edged this out, particularly in the training phase. The elastic net model of Zanon et al.^32^ also fell short of the accuracy of our model, underlining the effectiveness of our methodology. These comparisons underscore the high predictive capability of our model, which could be attributed to its comprehensive analytical approach, potentially making it a more reliable tool for early detection and management of diabetes.

Our study demonstrated a significant association between various clinical symptoms, demographic features, and patients' knowledge of diabetes in predicting the onset of the disease. Overall, our findings indicate a good predictive performance for type 2 diabetes and suggest that incorporating clinical symptoms, demographic features, and knowledge of diabetes can improve the accuracy of predictive models for type 2 diabetes. Therefore, it is imperative to raise awareness of diabetes risk factors, promote healthy lifestyles, and emphasize the importance of early diagnosis and treatment. Integrating these approaches into public health campaigns can help mitigate the prevalence of diabetes and its complications. In future studies, we intend to expand the validation of our model to encompass a more diverse range of populations. This, however, is dependent on obtaining datasets that accurately represent these groups. Through this endeavor, we aimed to strengthen the generalizability and widespread applicability of our diabetes detection model.

## AUTHOR CONTRIBUTIONS

**Taiwo Adetola Ojurongbe**: Conceptualization; data curation; investigation; project administration; resources; supervision; writing—review and editing. **Habeeb Abiodun Afolabi**: Conceptualization; investigation; methodology; project administration; resources; visualization; writing—original draft; writing—review and editing. **Adesola Oyekale**: Data curation; investigation; resources; writing—review and editing. **Kehinde Adekunle Bashiru**: Investigation; writing—review and editing. **Olubunmi Ayelagbe**: Conceptualization; data curation; investigation; project administration; resources; supervision; writing—review and editing. **Olusola Ojurongbe**: Investigation; project administration; supervision; writing—review and editing. **Saddam Akber Abbasi**: Investigation; writing—review and editing. **Nurudeen A. Adegoke**: Conceptualization; formal analysis; investigation; methodology; project administration; resources; software; supervision; validation; visualization; writing—review and editing.

## CONFLICT OF INTEREST STATEMENT

Authors declare no conflict of interest.

## TRANSPARENCY STATEMENT

The lead author Nurudeen A. Adegoke affirms that this manuscript is an honest, accurate, and transparent account of the study being reported; that no important aspects of the study have been omitted; and that any discrepancies from the study as planned (and, if relevant, registered) have been explained.

## Supporting information

Supporting information.

## Data Availability

The data supporting the findings of this study are available upon reasonable request from the corresponding author.
