# Supplementary material for: Predictive model for early detection of type 2 diabetes using patients' clinical symptoms, demographic features, and knowledge of diabetes
Source: Health Sci Rep. 2024 Jan 25;7(1):e1834. doi: 10.1002/hsr2.1834 (PMC10808992; doi:10.1002/hsr2.1834)
Supplement: Supplementary file 1 — Supporting information. [file HSR2-7-e1834-s001.docx]

**Supplementary Tables 1 and 2**

**Supplementary Table 1**. List of clinical variables used in the study and their units. Further details on how the biochemical and clinical samples were obtained are provided below.

| **S/N** | **Clinical symptom** | **Abbreviation** | **Unit** |
| --- | --- | --- | --- |
| 1 | Body weight | BW | Kg |
| 2 | Height | H | M |
| 3 | Body Mass Index | BMI | $\frac{Kg}{M^{2}}$ |
| 4 | Apolipoprotein C-III | APO-CIII | $\frac{mg}{dL}$ |
| 5 | Systolic Blood Pressure | SysBP | mmHg |
| 6 | Diastolic Blood Pressure | DiaBP | mmHg |
| 7 | Waist Circumference | WC | Cm |
| 8 | Hips Circumference | HC | Cm |
| 9 | Waist-Hips-Ratio | WHR |  |
| 10 | Carbohydrates counts | CHO | $\frac{mmol}{l}$ |
| 11 | Triglyceride | TG | $\frac{mmol}{l}$ |
| 12 | High-density lipoprotein | HDLC | $\frac{mmol}{l}$ |
| 13 | Low-density lipoprotein | LDLC | $\frac{mmol}{l}$ |
| 14 | Atherogenic indices of plasma | AIP |  |
| 15 | Cardiac risk ratio | CRR |  |
| 16 | Non-high density lipoprotein | Non.HDL |  |
| 17 | Atherogenic Coefficient | atherogenic coefficient |  |
| 18 | Malondialdehyde | MDA | $\frac{nmol}{ml}$ |
| 19 | Superoxide dismutase | SOD | $\frac{umol}{ml}$ |
| 20 | Catalase | Catalase | $\frac{pg}{ml}$ |
| 21 | Fasting Blood Sugar | FBS |  |
| 22 | Haemoglobin A1C | HbA1C | $\frac{mmol}{l}$ |

**Measurement of Biochemical and Clinical Parameters.**

After a minimum 12-hour fast, blood samples were collected for biochemical analyses. Peripheral venous blood samples (8 ml) from each participant were aliquoted into bottles labelled plain, EDTA, and fluoride oxalate. To obtain the serum, the samples were centrifuged at 3000 rpm for 10 min after they were allowed to clot. Plasma samples were separated by spinning at 1500 rpm for 30 min at 4 °C.

**Anthropometric Measurements.** Using a stadiometer, participants' height without shoes was measured to the nearest inch. A digital scale (SECA North America, Chino, CA, USA) was used to measure weight to the nearest kilogram. For the analysis, weight and height were both converted to kilograms. Underweight people (18.5 kg/m2), normal people (18.5 to 25 kg/m2), overweight people (25 to 30 kg/m2), and obese people (>30 kg/m2) were categorized based on self-reported and measured BMI ^1–4^.

**Blood pressure measurements**. A mercury sphygmomanometer was used to obtain two blood pressure (BP) readings, which were rounded up to the nearest 2 mmHg. After a 5-minute break between measurements, two readings were collected from each subject’s left and right arms. Systolic and diastolic blood pressures were calculated as the average of these values.

**Biochemical Analysis.** Enzymatic hydrolysis and oxidation were used to assess the fasting blood glucose, total cholesterol, HDL cholesterol, and TG levels ^5^. The Friedewald equation was used to determine the LDL cholesterol levels ^6^. ELISA kits (Cloud-Clone Corp., Houston, TX, USA) were used to measure serum malondialdehyde (MDA), catalase, and superoxide dismutase (SOD).

**Supplementary Table 2:** Baseline characteristics of patients in the training and test sets.

| **Predictor** | **Training set (N=312)** | **Test set (N=132)** | **Total (N=444)** | **p value** |
| --- | --- | --- | --- | --- |
| **Response** |  |  |  | 0.63 |
| No | 130 (41.7%) | 59 (44.7%) | 189 (42.6%) |  |
| Yes | 182 (58.3%) | 73 (55.3%) | 255 (57.4%) |  |
| **Obese** |  |  |  | 0.32 |
| No | 162 (51.9%) | 76 (57.6%) | 238 (53.6%) |  |
| Yes | 150 (48.1%) | 56 (42.4%) | 206 (46.4%) |  |
| **Are you on any medication for diabetes** |  |  |  | >0.99 |
| No | 141 (45.2%) | 59 (44.7%) | 200 (45.0%) |  |
| Yes | 171 (54.8%) | 73 (55.3%) | 244 (55.0%) |  |
| **Age** |  |  |  | 0.50 |
| Median (Range) | 40.000 (21.000, 68.000) | 41.000 (21.000, 68.000) | 40.000 (21.000, 68.000) |  |
| **Sex** |  |  |  | 0.09 |
| Female | 247 (79.2%) | 94 (71.2%) | 341 (76.8%) |  |
| Male | 65 (20.8%) | 38 (28.8%) | 103 (23.2%) |  |
| **BW** |  |  |  | 0.34 |
| Median (Range) | 71.000 (48.000, 95.000) | 71.000 (48.000, 95.000) | 71.000 (48.000, 95.000) |  |
| **Height** |  |  |  | 0.43 |
| Median (Range) | 1.590 (1.300, 1.860) | 1.600 (1.450, 1.860) | 1.600 (1.300, 1.860) |  |
| BMI |  |  |  | 0.20 |
| Median (Range) | 27.850 (17.760, 43.790) | 26.515 (17.760, 42.220) | 26.950 (17.760, 43.790) |  |
| APO.CIII |  |  |  | 0.08 |
| Median (Range) | 9.430 (1.400, 80.480) | 10.040 (1.400, 34.680) | 9.600 (1.400, 80.480) |  |
| Sys.Bp |  |  |  | 0.61 |
| Median (Range) | 128.000 (90.000, 240.000) | 128.000 (91.000, 187.000) | 128.000 (90.000, 240.000) |  |
| Dia.Bp |  |  |  | 0.39 |
| Median (Range) | 82.000 (56.000, 127.000) | 80.000 (56.000, 115.000) | 81.500 (56.000, 127.000) |  |
| Hypertensive status |  |  |  | 0.65 |
| Hypertensive | 109 (34.9%) | 44 (33.3%) | 153 (34.5%) |  |
| Normal | 124 (39.7%) | 49 (37.1%) | 173 (39.0%) |  |
| Pre hypertensive | 79 (25.3%) | 39 (29.5%) | 118 (26.6%) |  |
| Hypertensive Group |  |  |  | 0.83 |
| Hypertensive | 109 (34.9%) | 44 (33.3%) | 153 (34.5%) |  |
| Non hypertensive | 203 (65.1%) | 88 (66.7%) | 291 (65.5%) |  |
| WC |  |  |  | 0.93 |
| Median (Range) | 86.710 (62.660, 126.710) | 85.660 (59.660, 115.710) | 86.230 (59.660, 126.710) |  |
| HC |  |  |  | 0.95 |
| Median (Range) | 97.650 (70.650, 136.270) | 96.270 (72.220, 125.270) | 96.650 (70.650, 136.270) |  |
| WHR |  |  |  | 0.90 |
| Median (Range) | 0.900 (0.830, 0.930) | 0.900 (0.830, 0.930) | 0.900 (0.830, 0.930) |  |
| CHO |  |  |  | 0.78 |
| Median (Range) | 4.790 (2.630, 7.290) | 4.780 (2.630, 7.130) | 4.790 (2.630, 7.290) |  |
| TG |  |  |  | 0.84 |
| Median (Range) | 1.370 (0.170, 3.580) | 1.325 (0.170, 3.580) | 1.350 (0.170, 3.580) |  |
| HDL.C |  |  |  | 0.39 |
| Median (Range) | 1.130 (0.410, 2.280) | 1.140 (0.410, 2.190) | 1.130 (0.410, 2.280) |  |
| LDL.C |  |  |  | 0.68 |
| Median (Range) | 3.110 (1.120, 5.290) | 3.110 (1.120, 5.220) | 3.110 (1.120, 5.290) |  |
| LDL.HDL |  |  |  | 0.5 |
| Median (Range) | 2.970 (0.580, 11.120) | 2.990 (0.540, 9.150) | 2.970 (0.540, 11.120) |  |
| AIP |  |  |  | 0.70 |
| Median (Range) | 0.080 (-0.860, 0.670) | 0.080 (-0.830, 0.670) | 0.080 (-0.860, 0.670) |  |
| CRR |  |  |  | 0.50 |
| Median (Range) | 4.580 (1.760, 13.800) | 4.580 (1.670, 12.290) | 4.580 (1.670, 13.800) |  |
| Non.HDL |  |  |  | 0.83 |
| Median (Range) | 3.760 (1.240, 6.130) | 3.760 (1.240, 6.120) | 3.760 (1.240, 6.130) |  |
| Atherogenic coefficient |  |  |  | 0.50 |
| Median (Range) | 3.580 (0.760, 12.800) | 3.580 (0.670, 11.290) | 3.580 (0.670, 12.800) |  |
| MDA |  |  |  | 0.49 |
| Median (Range) | 0.730 (0.100, 1.680) | 0.760 (0.100, 1.680) | 0.730 (0.100, 1.680) |  |
| SOD |  |  |  | 0.39 |
| Median (Range) | 132.720 (23.080, 310.150) | 141.650 (23.080, 310.150) | 138.025 (23.080, 310.150) |  |
| Catalase |  |  |  | 0.33 |
| Median (Range) | 33.460 (17.400, 54.430) | 32.040 (17.400, 70.910) | 33.460 (17.400, 70.910) |  |
|  |  |  |  |  |
|  |  |  |  |  |
| HbA1C |  |  |  | 0.68 |
| Median (Range) | 8.330 (3.870, 14.050) | 8.500 (3.870, 14.050) | 8.480 (3.870, 14.050) |  |
| Eyes retinopathy |  |  |  | 0.66 |
| No | 300 (96.2%) | 125 (94.7%) | 425 (95.7%) |  |
| Yes | 12 (3.8%) | 7 (5.3%) | 19 (4.3%) |  |
| Kidneys proteinuria or nephropathy |  |  |  | 0.65 |
| No | 298 (95.5%) | 128 (97.0%) | 426 (95.9%) |  |
| Yes | 14 (4.5%) | 4 (3.0%) | 18 (4.1%) |  |
| Nerves or feet neuropathy |  |  |  | >0.99 |
| No | 286 (91.7%) | 121 (91.7%) | 407 (91.7%) |  |
| Yes | 26 (8.3%) | 11 (8.3%) | 37 (8.3%) |  |
| Heart attack or blocked heart arteries |  |  |  | 0.97 |
| No | 288 (92.3%) | 121 (91.7%) | 409 (92.1%) |  |
| Yes | 24 (7.7%) | 11 (8.3%) | 35 (7.9%) |  |
| Slowed digestion gastroparesis |  |  |  | 0.09 |
| No | 281 (90.1%) | 126 (95.5%) | 407 (91.7%) |  |
| Yes | 31 (9.9%) | 6 (4.5%) | 37 (8.3%) |  |
| Eating too much sugar and sweet foods is a cause of Diabetes |  |  |  | 0.15 |
| No | 21 (6.7%) | 15 (11.4%) | 36 (8.1%) |  |
| Yes | 291 (93.3%) | 117 (88.6%) | 408 (91.9%) |  |
| A common cause of diabetes is insulin resistance in the body |  |  |  | 0.23 |
| No | 19 (6.1%) | 13 (9.8%) | 32 (7.2%) |  |
| Yes | 293 (93.9%) | 119 (90.2%) | 412 (92.8%) |  |
| Diabetes is hereditary |  |  |  | 0.82 |
| No | 50 (16.0%) | 23 (17.4%) | 73 (16.4%) |  |
| Yes | 262 (84.0%) | 109 (82.6%) | 371 (83.6%) |  |
| medication is more important than diet and exercise to control diabetes |  |  |  | 0.37 |
| No | 89 (28.5%) | 44 (33.3%) | 133 (30.0%) |  |
| Yes | 223 (71.5%) | 88 (66.7%) | 311 (70.0%) |  |
| Diabetes often causes poor circulation |  |  |  | 0.72 |
| No | 259 (83.0%) | 107 (81.1%) | 366 (82.4%) |  |
| Yes | 53 (17.0%) | 25 (18.9%) | 78 (17.6%) |  |
| Cuts and wounds heal more slowly in diabetics |  |  |  | 0.72 |
| No | 83 (26.6%) | 38 (28.8%) | 121 (27.3%) |  |
| Yes | 229 (73.4%) | 94 (71.2%) | 323 (72.7%) |  |
| Diabetes can lead to decreased sensitivity of the hands, fingers and feet |  |  |  | 0.72 |
| No | 90 (28.8%) | 41 (31.1%) | 131 (29.5%) |  |
| Yes | 222 (71.2%) | 91 (68.9%) | 313 (70.5%) |  |
| Tremors and sweating are signs of high sugar in the blood |  |  |  | 0.37 |
| No | 105 (33.7%) | 51 (38.6%) | 156 (35.1%) |  |
| Yes | 207 (66.3%) | 81 (61.4%) | 288 (64.9%) |  |
| smoking and consumption of alcohol contribute to the complication of diabetes |  |  |  | 0.23 |
| No | 54 (17.3%) | 30 (22.7%) | 84 (18.9%) |  |
| Yes | 258 (82.7%) | 102 (77.3%) | 360 (81.1%) |  |

**References**

1. Skeie G, Mode NA, Henningsen MH, Borch KB. Validity of self-reported body mass index among middle-aged participants in the Norwegian Women and Cancer study. *Clinical Epidemiology*. Published online 2015. doi:10.2147/clep.s83839

2. Ikeda N. Validity of Self-Reports of Height and Weight among the General Adult Population in Japan: Findings from National Household Surveys, 1986. *Plos One*. 2016;11(2). doi:10.1371/journal.pone.0148297

3. Yazawa A, Inoue Y, Kondo N, et al. Accuracy of self‐reported weight, height and body mass index among older people in Japan. *Geriatrics &Amp; Gerontology International*. 2020;20(9). doi:10.1111/ggi.13971

4. Marshall N, Guild C, Cheng YW, Caughey AB, Halloran DR. Maternal superobesity and perinatal outcomes. *American Journal of Obstetrics and Gynecology*. 2012;206(5). doi:10.1016/j.ajog.2012.02.037

5. Balan R, Antczak A, Brethauer S, Zielenkiewicz T, Luterbacher JS. Steam Explosion Pretreatment of Beechwood. Part 1: Comparison of the Enzymatic Hydrolysis of Washed Solids and Whole Pretreatment Slurry at Different Solid Loadings. *Energies*. 2020;13(14). doi:10.3390/en13143653

6. Friedewald WT, Levy RI, Fredrickson DS. Estimation of the concentration of low-density lipoprotein cholesterol in plasma, without use of the preparative ultracentrifuge. *Clin Chem*. 1972;18(6):499-502.
